# The complete mitochondrial genome sequence of *Taxonus zhangi* Wei, 1997 (Hymenoptera: Tenthredinidae) with phylogenetic analysis

**DOI:** 10.1080/23802359.2021.1987173

**Published:** 2021-10-14

**Authors:** Min Xu, Beibei Tan, Meicai Wei, Gengyun Niu

**Affiliations:** College of Life Sciences, Jiangxi Normal University, Nanchang, Jiangxi, China

**Keywords:** Mitochondrial genome, phylogeny, Allantinae, Taxonina, Allantina

## Abstract

The complete mitochondrial genome of *Taxonus zhangi* was 16,002 bp in size, comprises 13 protein-coding genes (PCGs), two rRNA genes, 22 tRNA genes, and large non-coding A + T region. The phylogenetic result confirms the monophyly of Taxonina and Allantina, and also supports that Xenapateini is the sister group of Allantini which is composed of Taxonina and Allantina.

*Taxonus* Hartig, 1837 is the largest and complicated genus of Allantinae, Tenthredinidae. It was ever divided into several genera (Malaise 1963). Based on morphological studies, *Taxonus* and its relatives represent a distinct lineage, Taxonina, within Allantini of Allantinae, and Taxonina is a sister group of Allantina of Allantini (Wei and Nie 1998). More mitogenome data of the group are needed to confirm the systematic position of the *Taxonus* lineage within Allantinae and the monophyly of the complicated *Taxonus*. Here we report the mitochondrial genome of *Taxonus zhangi*.

The specimens of *T. zhangi* used for this study were captured at Jinghai parking lot, Jiuzhaigou National Natural Reserve, Jiuzhaigou County, Sichuan Province (33.17 N, 103.89 E) in July 2020. The freshly collected specimens were preserved immediately in absolute ethanol. The specimens were identified by Wei Meicai. Voucher specimen is deposited in the Asia Sawfly Museum, Nanchang (ASMN) (Meicai Wei, weimc@126.com) under the voucher number CSCS-Hym-MC0342. Whole genomic DNA was extracted from the thorax muscle of a female adult using the DNeasyR Blood &Tissue Kits (Qiagen, Valencia, CA). Genomic DNA was prepared in 150 bp paired-end libraries, tagged, and analyzed with the high-throughput Illumina Hiseq 4000 platform (Illumina, San Diego, CA), yielding a total of 100,546,654 raw reads (SRR14860554). It was further assembled by MitoZ (Meng et al. 2019) and verified by Geneious Prime 2019.2.1 (https://www.geneious.com). Annotations were generated using the MITOS web server (Bernt et al. 2013) and revised when necessary. The sequences were multiply aligned using MAFFT method in the TranslatorX server (Abascal et al. 2010). The phylogenetic tree under GTR model was conducted on IQTREE web (Trifinopoulos et al. 2016).

**Figure·1.**
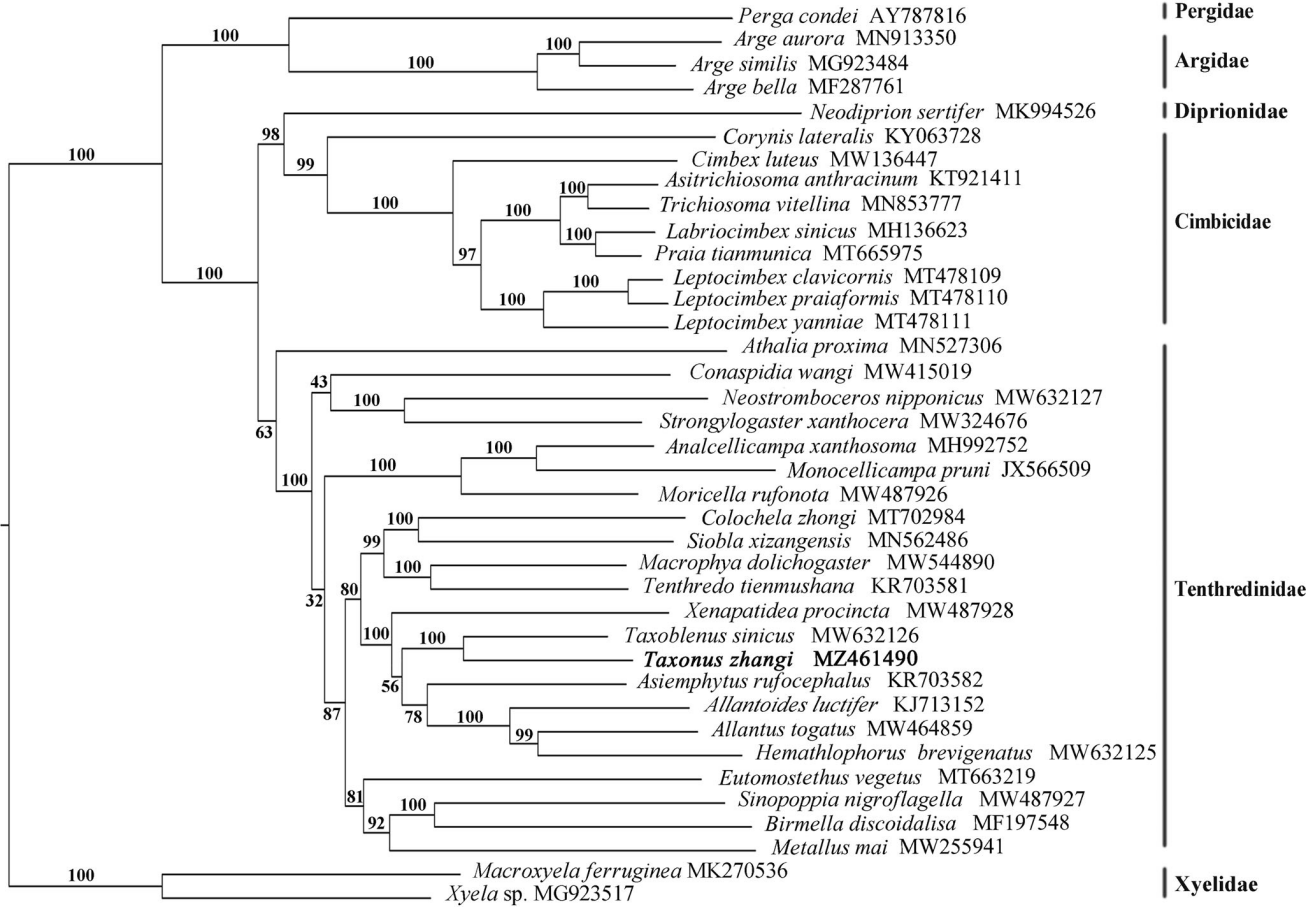
Phylogenetic tree based on the combination of 13 PCGs. Numbers at the left of nodes are bootstrap support values. The accession number of each species is indicated after the Latin name.

The sequence yield by MitoZ was 15,819 bp and contained 37 genes with a partial control region (CR). *Allantus togatus* (MW464859) and *Taxoblenus sinicus* (MW632126) were used as references with the mean depth of coverage across the sequences being 20,452 and 11,737, respectively, which prove the accuracy of the sequence generated by MitoZ. Reassembly using *trnI* and *trnQ* as references extended contigs and obtained a 454 bp overlap. Then, we obtained a control region of 879 bp in length manually. Furthermore, we used the 454 bp overlap as a reference to verify the reliability of the results. By consistently obtaining similar coverage of the assembly contigs, we were able to confirm the control region between *trnI* and *trnQ*.

The complete mitogenome of *T. zhangi* (GenBank accession number MZ461490) is 16,002 bp in length, containing 13 protein-coding genes (PCGs), 22 transfer RNA (tRNA) genes, two ribosomal RNA (rRNA) genes, and one large non-coding A + T region. The gene content, arrangement, and composition exhibited a typical insect mitogenome feature. Overall, the *T. zhangi* mitogenome has an A + T content of 81.2% (42.60% A, 11.40% C, 7.30% G, and 38.60% T), indicating significant A + T bias. All PCGs use ATN as a start codon (N, any nucleotide). The *nad4L* gene use TAG as a stop codon, *nad2* and *nad4* use a single T as stop codon, and the rests use TAA as a stop codon.

The phylogenetic tree was constructed based on mitochondrial genome sequences of 38 Tenthredinoidea species with two Xyelidae species as outgroup (Figure 1). Phylogenetic analysis indicated that *Taxonus zhangi* is a sister group of *Taxoblenus sinicus* and supports the monophyly of Taxonina. Furthermore, Taxonina (*T. zhangi* + *T. sinicus*) is a sister group of Allantina (*Asiemphytus*+(*Allantoides*+(*Allantus*+*Hemathlophorus*))), and *Xenapatidea* is a sister group of Allantina + Taxonina. This result supports well the morphological phylogeny of Allantinae except for the position of *Hemathlophorus* (Wei and Nie 1998). More samplings are needed to make clear the systematic position of *Hemathlophorus* and its relatives, such as *Athlophorus* and *Thaumatotaxonus*, within Tenthredinidae.

## Data Availability

The genome sequence data that support the findings of this study are openly available in GenBank of NCBI at https://www.ncbi.nlm.nih.gov under the accession number MZ461490. The associated BioProject, SRA, and BioSample numbers are PRJNA736586, SRR14860554, and SAMN19655083, respectively. All related files had been uploaded to figshare https://figshare.com/account/home#/projects/117060.
